# Accuracy of Spot Urine Albumin Creatinine Ratio With Respect to 24-Hour Urine Albumin for the Detection of Proteinuria in Antenatal Women With Preeclampsia: A Descriptive Study

**DOI:** 10.7759/cureus.39961

**Published:** 2023-06-05

**Authors:** Shraddha Rao, Savita Somalwar, Anuja Bhalerao, Vishal Raman

**Affiliations:** 1 Department of Obstetrics and Gynecology, NKP Salve Institute of Medical Sciences and Research Center, Nagpur, IND

**Keywords:** antenatal women, preeclampsia, proteinuria, 24 hour urine albumin, spot urine albumin creatinine ratio

## Abstract

Background

Among the hypertensive disorders of pregnancy, certain diseases like preeclampsia (PE) and eclampsia have the maximum impact on morbidity and mortality of the mother as well as the newborn. Proteinuria determination is used to assess renal damage in PE. There are several ways to evaluate proteinuria in pregnant women, but the gold standard remains the 24-hour urine albumin (24-h UA) excretion. Spot urine albumin creatinine ratio (UACR) can be used for rapid diagnosis of PE which is fast, reliable, and easy to use. Hence, our tertiary care center conducted the current study to assess the accuracy of spot UACR with 24-h UA for detecting proteinuria in antenatal women to diagnose PE and to evaluate the obstetric outcome in antenatal women with PE.

Methodology

A descriptive, cross-sectional study was conducted on 98 antenatal women diagnosed with PE. Urine albumin was done by dipstick method and the presence or absence of proteinuria was noted. Both, the 24-h urine sample and a random sample for spot UACR were sent for analysis.

Results

Spot UACR has more specificity than sensitivity along with a high negative predictive value for the detection of proteinuria. Additionally, significant proteinuria was associated with an increased rate of induced labor, a cesarean section in patients, lower mean gestational age at the time of delivery, lower birth weight, and increased rate of intrauterine fetal death.

Conclusion

The study concluded that spot UACR has more specificity than sensitivity along with a high negative predictive value for the detection of proteinuria and therefore, can be used for the diagnosis of proteinuria in women with PE. Hence, spot UACR is a reliable, faster, and more accurate method for the detection of proteinuria in PE and can be used for early diagnosis and timely management leading to a decrease in mortality and morbidity of the mother and the fetus.

## Introduction

About 10% of pregnant women experience hypertensive problems [1,2]. Among the hypertensive disorders of pregnancy, certain diseases like preeclampsia (PE) and eclampsia have the maximum impact on morbidity and mortality of the mother as well as the newborn. PE, which affects 2%-8% of pregnancies overall, is a significant factor in maternal death [3]. Pregnancy hypertensive disorders include chronic hypertension, gestational hypertension, PE, and eclampsia. PE is defined as a specific disease related to pregnancy in which there is the occurrence of hypertension and significant proteinuria in a previously healthy woman on or after the 20th week of gestation and occurs in about 2%-8% of pregnancies. Additionally, proteinuria in PE is not essential to diagnose but is related to disease severity, and fetal outcomes as substantial levels of proteinuria consisting of ≥300 mg/24h are linked to it [4]. This disease begins post 20 weeks of pregnancy and if it is not dealt with timely, it might result in tonic-clonic seizures, which is known as eclampsia [5,6]. It can appear as delayed as four to six weeks postpartum. In PE, there is widespread vascular endothelial malfunction and vasospasm.

The exact cause of PE remains unknown however, this disease begins earlier in gestation. The pathophysiology is such that changes occur which gain momentum across increasing gestational periods and become apparent clinically. It ultimately results in the involvement of various organs involving the kidney, brain, and heart. In maternal circulation, there is a variance between the anti-angiogenic factors and pro-angiogenic factors. The maternal kidney is particularly vulnerable to injury as a result of this imbalance [7].

Proteinuria determination is used to assess renal damage in PE. With advancing pregnancy, proteinuria can get exacerbated. As per contemporary recommendations, proteinuria is not essential for the detection of PE [8,9]. It is presumed that PE can occur prior to the glomerular capillary endothelial damage becoming drastic enough to initiate proteinuria. However, it cannot be denied that, as an exhaustive index reflecting numerous factors in circulation, the diagnosis of PE with renal impairment is now more sensitive and specific because of urine protein levels [10]. More importantly, it has been demonstrated that proteinuria is related to worse pregnancy outcomes [11,12]. But there is not any consensus about the significance of proteinuria in women suffering from pregnancy with hypertensive disorders.

However, one of the pillars of antenatal care is screening for PE with regular BP checks and urine tests for proteinuria. Spot UACR and dipstick method are other ways to evaluate proteinuria in pregnant women, but the gold standard remains the 24-hour urine albumin (24-h UA) excretion. Although the 24-h urine test is the gold standard test, it has its own limitations as it is time-consuming. The dipstick method, which is used as a routine bedside test to detect proteinuria, is unreliable due to its high false negatives and false positives, and its dependence on maternal hydration status [13-16].

Spot urine albumin creatinine ratio (UACR) can be used for rapid diagnosis of PE which is fast, reliable, and easy to use. Thus, a quicker and yet more accurate test would offer an immediate prognosis resulting in a reduction in maternal and perinatal mortality. Numerous researchers have looked into the clinical importance of spot UACR during pregnancy. Some studies have shown accuracy when compared with 24-h UA [17-20] while some studies have shown that spot UACR is not as accurate as 24-h UA [21,22], and yet there is no consensus opinion. Currently, spot UACR is not used as a routine test to evaluate proteinuria in women with hypertensive disorders. Hence, our tertiary care center conducted the current study to assess the accuracy of spot UACR with 24-h UA for detecting proteinuria in antenatal women to diagnose PE. To evaluate the obstetric outcome in antenatal women with PE.

## Materials and methods

A descriptive, cross-sectional study was conducted at a tertiary care hospital attached to a medical college in the Department of Obstetrics and Gynecology from January 2021 to December 2022. Ninety-eight antenatal women diagnosed with PE and admitted to the ward and labor room were considered for the study. Antenatal women diagnosed with PE delivering after 24 hours or more of admission with gestational age ≥28 weeks and antenatal women diagnosed with PE willing to deliver at the study place were included in the study whereas, women with chronic hypertension, renal disease, diabetes mellitus, thyroid, and other endocrine disorders, liver disease, heart disease, urinary tract infection, and hydatidiform mole were excluded from the study.

Institutional ethics committee approval was taken with reference number 102/2021 along with written informed consent following which patients were selected as per inclusion criteria. A convenient sampling technique was employed. Patients with raised BP and proteinuria, ≥2+ by dipstick method diagnosed with PE were included in the study. Patients in whom blood pressure was in the non-severe range, and without proteinuria, but with warning signs and symptoms or deranged lab parameters were also included in this study. Based on the blood pressure values patients were accordingly classified as those having severe and non-severe PE.

Detailed history regarding demographic details, menstrual history, and obstetric history was obtained. Risk factors of PE such as age, gravidity, parity, and past history of hypertensive disorders were recorded. A thorough general, cardiovascular and respiratory, and obstetrical examination was performed, and blood pressure was measured using a sphygmomanometer. In women with blood pressure more than 140 systolic and more than 90 diastolic but less than 160 systolic and less than 110 diastolic blood pressure (DBP) the test was repeated after four hours. In women with blood pressure more than 160 systolic and 110 DBP it was repeated within minutes. Urine albumin was done by dipstick method and the presence or absence of proteinuria was noted. If proteinuria was present the grade, i.e., trace, +1, +2, +3, and +4 was noted, and ≥2+ was considered as significant proteinuria, and trace or +1 was considered non-significant proteinuria.

Those women with urinary tract infections on urine microscopy were excluded. Those patients who had at least 24 h till delivery were included in the study in whom spot UACR was done on admission in a 50 mL urine container for laboratory analysis for spot UACR. All the patients were subsequently asked to collect a urine sample for the next 24h. Patients were asked to discard the first void and collect the 24-h sample thereafter in a large container. Out of the total sample, 5mL of urine was sent to the laboratory for estimation of proteinuria and the following formula was used to calculate the proteinuria. Total 24-h UA excretion = Urine protein concentration (mg/dL) × 24-h urine volume in mL/100. Both the 24-h urine sample and a random sample for spot UPCR were sent for analysis. Analysis of both samples was done using Siemens Dimensions RxL Max, which is fully automated, and the maternal and fetal outcomes were noted.

Mean and standard deviation were used to present quantitative data. Using the findings of the normality test, an unpaired t-test is used to compare the research groups. A frequency and percentage table were used to convey qualitative data. Fisher, student, and Chi-square tests were used to determine whether there was any association between the study groups. A “p” value of 0.05 or less was considered significant.

## Results

A hospital-based descriptive, cross-sectional study was conducted to measure the accuracy of spot UACR with respect to 24-h UA for diagnosis of proteinuria in antenatal women with PE and the sample size was 98. The majority of patients (63.3%) belonged to the 20-25 age group, followed by the 26-30 age group (27.5%) and the over-30 age group (9.2%). The patient's average age was 25.27±3.61 years.

When considering BMI 23 (23.5%) and 14 (14.3%) patients, respectively, were overweight and obese, and 61 (62.2%) of the patients had BMIs that were within the normal range. The patients' average BMI was 25.27±3.61 kg/m^2^. Thirty-three (33.6%) patients were Nullipara while 25 (25.5%) were para 1. Twenty-four (24.48%) patients were para 2. Eleven (11.2%) and five (5.1%) patients were para 3 and ≥ para 4, respectively. Furthermore, the gestational age in 51 (52.1%) patients was in the range of 36-39.6 weeks. The gestational age was in the range of 32-35.6 weeks for 44 (44.8%), while in three (3.1%) patients was in the range of 28-31.6 weeks. The mean gestational age of patients was 36.15±2.53 weeks. Additionally, the mean systolic blood pressure (SBP) value in patients was 150.12±8.95 mmHg while the mean DBP value was 101.20±6.12 mmHg. Furthermore, 59 (60.2%) patients had non-severe PE while 39 (39.8%) patients had severe PE.

In contrast to 84 (85.7%) patients who had substantial proteinuria (≥ 300 mg/day), 14 (14.3%) patients had no proteinuria (<300 mg/day). Patients' mean 24-h UA was 616.64± 371.30 mg/day as shown in Table 1.

**Table 1:** Distribution of patients according to 24-h UA. 24-h UA = 24-hour urine albumin

24-hour urine albumin	N	%
<300 mg/day	14	14.3
≥300 mg/day	84	85.7
Total	98	100

Sixteen (16.3%) patients had spot UACR<0.3 mg/dL while 82 (83.7%) patients had spot UACR ≥ 0.3 mg/dL. The mean spot UACR of patients was 0.797±0.357 mg/dL as demonstrated in Table 2.

**Table 2:** Distribution of patients according to spot UACR UACR = Urine albumin creatinine ratio

Spot UACR	N	%
<0.3 (mg/dL)	16	16.3
≥0.3 (mg/dL)	82	83.7
Total	98	100

The urinary dipstick grading was 3+ (positive) in 74 (75.5%) patients and 2+ (positive) in 24 (24.5%) patients. Significant proteinuria, that is≥+2 was seen in 84 patients (85.7%). While 14 patients (14.3%) did not have significant proteinuria, five (5.1%) had +1 and which was considered non-significant proteinuria and nine (9.2%) had negative findings as demonstrated in Table 3.

**Table 3:** Distribution of patients according to urinary dipstick grading

Urinary dipstick grading	N	%
+3	74	75.5
+2	10	10.2
+1	5	5.1
Negative	9	9.2
Total	98	100

The majority of patients with Spot UACR ≥ 0.3 mg/dL (80 out of 82 patients; 97.5%) had 24-h UA ≥ 300 mg/day as shown in Table 4.

**Table 4:** Validity of spot UACR in prediction of significant proteinuria compared to 24-h UA UACR = Urine albumin creatinine ratio; 24-h UA = 24-hour urine albumin

Spot UACR (mg/dl)	24-h UA (mg/day)				Total
	<300		≥300		
	N	%	N	%	
<0.3	12	75	4	25	16
≥0.3	2	2.5	80	97.5	82
Total	14	14.3	84	85.7	98

The positive predictive value (PPV) and negative predictive value (NPV) were respectively 75.00% and 97.56%, while the sensitivity and specificity were 85.71% and 95.24% as shown in Table 5.

**Table 5:** Sensitivity and specificity of spot UACR UACR = Urine albumin creatinine ratio; PPV = Positive predictive value; NPV= Negative predictive value

Spot UACR	Value	95% CI
Sensitivity	85.71%	57.19% to 98.22%
Specificity	95.24%	88.25% to 98.69%
PPV	75.00%	52.96% to 88.88%
NPV	97.56%	91.72% to 99.31%

Spot UACR had better sensitivity (85.71% versus. 81.25%), specificity (95.24% versus. 86.59%), PPV (75.00% versus. 74.57%), and NPV (97.56% versus. 54.17%) as described in Table 6.

**Table 6:** Comparison of spot UACR and urinary dipstick grading UACR = Urine albumin creatinine ratio.

Statistic	Spot UACR	Urinary dipstick grading
Sensitivity	85.71%	81.25%
Specificity	95.24%	86.59%
PPV	75.00%	74.57%
NPV	97.56%	54.17%

**Figure 1:**
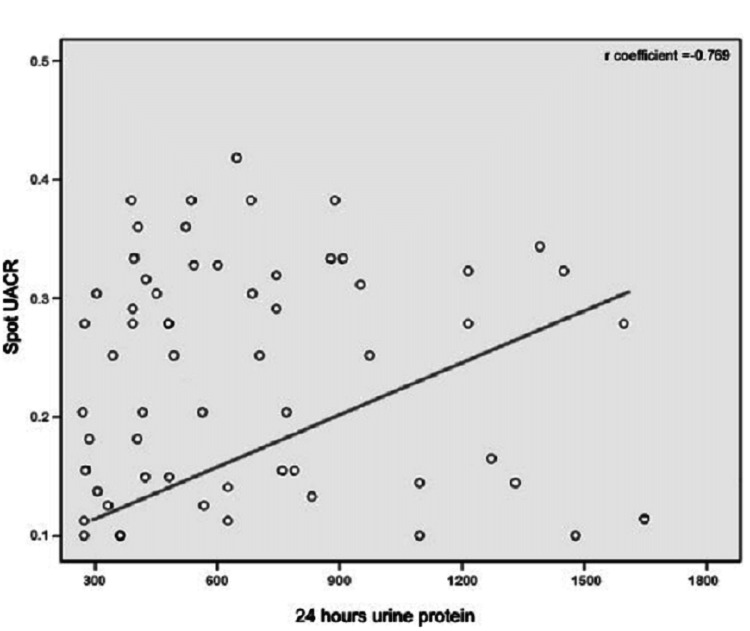
Correlation between spot UACR and 24-h UA level UACR = Urine albumin creatinine ratio; 24-h UA = 24-hour urine albumin

The correlation between 24-h UA and Spot UACR showed that with a correlation coefficient (r) of 0.769, there was a strong positive statistical association between 24-h UA and Spot UACR as shown in Figure 1. The ROC curve's area under it had a value of 0.97 (95% CI: 0.94-0.99; p<0.05). For diagnosing substantial proteinuria (300 mg/day), the ROC curve provided the most discriminant value for spot UACR of 290.4 mg/g, which provided the best sensitivity, specificity, PPV, and NPVs. A sensitivity of 97.87% and a specificity of 80.24% were obtained from the spot UACR at 290.4 mg/g.

According to the student t-test (p<0.05), a significantly higher percentage of patients with severe proteinuria underwent induced labor (78.6%) than patients with no proteinuria (57.2%) or non-significant proteinuria. In a similar manner, a larger proportion of patients with substantial proteinuria (76.2%) than those without it (23.8%) received cesarean delivery, according to a Student's t-test (p<0.05). Both groups had comparable rates of interruption (7.1% versus 4.8%), as well as post-partum hemorrhage (PPH) (14.2% and 15.5%). In our investigation, one patient with substantial proteinuria (1.2%) passed away as described in Table 7.

**Table 7:** Distribution of patients according to maternal outcome PPH = Postpartum haemorrhage; ICU = Intensive Care Unit

Maternal Outcome	No significant proteinuria (n=14)		Significant proteinuria (n=84)		p Value
	N	%	N	%	
Type of labour					<0.05
Spontaneous	6	42.8%	18	21.4%	
Induced	8	57.2%	66	78.6%	
Mode of Delivery					<0.05
Vaginal	5	35.7%	20	23.8%	
Caesarean	9	64.3%	64	76.2%	
Complications					
PPH	2	14.2%	11	13.1%	>0.05
Abruption	1	7.1%	4	4.8%	>0.05
Renal failure	1	7.1%	3	3.6%	>0.05
Requiring ICU Admission	0	-	2	2.4%	>0.05
Maternal Mortality	0	-	1	1.2%	>0.05

The mean gestational age at delivery was substantially lower in patients with considerable proteinuria (37.85±1.81 weeks) than it was in individuals with no proteinuria (39.14±0.36 weeks) or non-significant proteinuria. Patients with severe proteinuria had substantially lower mean fetal birth weights (2.37±0.42 kg) than patients without proteinuria (2.61±0.41 kg) or non-significant proteinuria. When compared to seven (15.2%) patients without proteinuria, 46 (54.8%) patients with substantial proteinuria gave birth to neonates with low birth weight (<2.5 kg), and this difference was statistically significant. Both groups experienced a similar rate of NICU admissions and APGAR scores of <7 at 5 minutes. Patients with severe proteinuria had a significantly increased risk of intrauterine fetal death (IUFD) (2.38% p<0.05). In the present investigation, one (1.2%) infant with severe proteinuria died as described in Table 8.

**Table 8:** Distribution of patients according to fetal outcome GA - Gestational Age; LBW - Low Birth Weight; IUFD - Intrauterine fetal death; NICU - Neonatal Intensive Care Unit

Fetal outcome	No significant proteinuria (n=14)		Significant proteinuria (n=84)		P-value
	N	%	N	%	
GA at Delivery	39.14	0.36	37.85	1.81	<0.05
Fetal birth weight	2.61	0.41	2.37	0.42	<0.05
LBW (<2.5 kg)	7	15.2%	46	54.8%	<0.05
NICU Admission	2	14.2%	12	14.3%	>0.05
APGAR <7 at 5 mins	3	21.4%	21	25%	>0.05
IUFD	0	-	2	2.38%	<0.05
Neonatal death	0	-	1	1.2%	>0.05

## Discussion

The current study was carried out from January 2021 to December 2022, among patients admitted in the labor room and obstetrics ward at a tertiary care hospital and referral hospital. The primary objective of the study was to measure the accuracy of spot UACR with respect to 24-h UA for diagnosis of proteinuria in antenatal women with PE and the secondary objective was to evaluate the obstetric outcome in antenatal women with PE. The study enrolled 98 women having singleton pregnancies with PE. The patient's average age was 25.27±3.61 years. According to the aforementioned research, 61 (62.2%) of the patients in the current study had BMIs that were within the normal range, whereas 23 (23.5%) and 14 (14.3%) of the patients were overweight or obese, respectively. The patients' average BMI was 25.27±3.61 kg/m^2^. In a previous study by Mahesh et al., the average age was 23.73 years and the average BMI was 20.19 kg/m^2^ [18].

Primigravida is a known risk factor for causing PE. In the present study, the number of patients that were nullipara involved 33 (33.6%), para gravida 1 involved 25 (25.5%), para gravida 2 consisted of 24 (24.48%), followed by para 3 including 11 (11.2%), and more than or equal to para 4 involving five (5.1%). In a similar study by Modak et al. in 2019, the maximum number of patients involved was primigravida which consisted of eight (53.33%) patients, followed by para 2 which involved four (26.67%) patients, and para 3 which involved three (20%) patients [19].

In the present study, the mean SBP was found to be 150.12±8.95 and the mean DBP was found to be 101.20±6.12, which corresponds with a study given by Amin et al. and Mahesh et al. that demonstrated mean SBP of 152±18.2 range between 132-178 and 155.26±10.08, respectively, and mean DBP of 96.4±11.3 ranging between 78-116 and 102.11±7.04, respectively [18,23]. In contrast with the above study given by Modak et al., the mean SBP and DBP were 117.6 and 72.4, respectively [19]. In this study, the maximum number of patients were in the non-severe PE group 59 (60.2%) and the rest were in the severe PE 39 (39.8%). in congruous with the above-mentioned studies, Nischintha et al. reported a maximum number of patients in the non-severe PE group (73.3%) and (26.7%) in the severe PE group [24]. Additionally, in contrast with the present study, previous research by Amin et al. reported the majority of the woman were in the stage of severe eclampsia 43 (42.2%) and only 22 (21.6%) were in the non-severe PE group [23].

In the present study, the mean of 24-h UA was found to be 616.64±371.30 involving 14 (14.3%) patients in <300 mg/day and 28 (27.5%) in >300 mg/day. Similarly, in a previous study given by Amin et al. in 2014, the mean was found to be 1446±1242 mg/day involving 24 (23.5%) in <300 mg/day and 28 (27.5%) in >300 mg/day [23]. Additionally, the mean 24-h UA found in studies are given by Jan et al. and Mahesh et al. reported 1,070±800 mg/day and 1,284.29±844.40, respectively [18,25]. The studies concluded that the sensitivity and specificity of 24-h UA is best but this collection method is tedious and not possible in case of an emergency when there is no time.

In the present study, the spot UACR was found to be 0.797 ±0.357 mg/dL, which is similar to a study given by Jan et al. that demonstrated a UACR of 576.5±211.4 mg/g [25]. Additionally, studies given by Amin et al. and Hossain et al. demonstrated values of 1.09±0.86 mg/dL ranging between 0.1-3.41 and 1.14±1.87 mg/dL ranging between 0.03 and 9.73, respectively [17,23]. In contrast with the above-mentioned studies, the study given by Mahesh et al. demonstrated a very high mean of UACR which is 1,812.44±1,162.58 mg/g [18]. An alternate test to 24-h urine protein collection is spot UACR which is difficult, time-consuming inconvenient, and prone to error because of inaccuracy in timings and/or incompleteness. The approach of detecting proteinuria is quicker, within safe bounds, and helps with diagnosis and early treatment, resulting in better fetomaternal outcomes.

In the present study, 74 (75.5%) were found to be positive (+3) by urinary dipstick grading which is similar to a study given by Mahesh et al. that reported 53 patients positive with urinary dipstick grading [18] whereas contrast with the above-mentioned studies Amin et al. reported a very less number of positive (+3) patients 10 (9.8%) [23]. In the present study, the sensitivity of 85.71%, and specificity of 95.24% validates the spot UACR in the prediction of significant proteinuria which is similar to a study given by Mahesh et al. and Modak et al. that demonstrated sensitivity of 95% and 80%, respectively, and specificity of 80% and 94.06%, respectively [18,19]. In contrast with the above-mentioned studies, the study given by Amin et al. reported high sensitivity of 89.7% with low specificity of 54.2% [23]. In the present study, spot UACR demonstrated high sensitivity (85.71%) and specificity (95.24%) which makes it valid in predicting significant proteinuria as compared to urinary dipstick grading. A similar study by Mahesh et al. reported high sensitivity (95%) and specificity (80%) for spot UACR as compared to urinary dipstick grading [18]. Hence, based on the studies it can be concluded that spot UACR is a reliable, quick, and accurate method for identifying proteinuria in PE. This method may enable better management of preeclamptic women, lowering maternal and perinatal morbidity and mortality, particularly in low-resource settings and developing nations like India.

The correlation coefficient (r) in the current study was 0.769, and the p-value was 0.0001, which demonstrated a strong correlation between spot UACR and 24-h urinary protein level with statistically significant results. Similarly, the study given by Demirci et al. described an r-value of 0.758 demonstrating a good correlation with statistically significant results [26]. Additionally, Kayatas et al., reported an r-value of 0.828 demonstrating a strong correlation [22]. Furthermore, Sarkar et al., reported an r-value of 0.98 demonstrating a strong correlation with statistically significant results [20]. Whereas in some previous studies, poor correlation was observed such as 0.56 and 0.4 [22].

In the present study, the distribution of patients according to maternal outcomes consisted of spontaneous labor involving 18 (21.4%) patients, induced labor 66 (78.6%) patients, vaginal delivery 20 (23.8%) patients, cesarean delivery 64 (76.2%) patients, postpartum hypertension 11 (13.1%) patients, abruption four (4.8%) patients, renal failure three (3.6%) patients, requiring Intensive Care Unit (ICU) admission two (2.4%) patients, and maternal mortality one (1.2%) patient. Similarly, a study given by Cheung et al. reported maternal outcomes that involved severe hypertension involving 45 (47%) patients, renal insufficiency six (6%), and admission to ICU [27]. Hence, in conclusion, the most common maternal outcomes reported were induced labor and severe hypertension.

In the present study, the fetal outcomes consisted of gestational age at delivery involving 37.85 (1.81%) weeks, fetal birth weight of 2.37 kg (0.42%), low birth weight (<2.5 kg) of 46 (54.8%) kg, neonatal ICU admission involving 12 (14.3%) patients, APGAR <7 at 5 mins involved 21 (25%), IUFD included 17 (20.2%) patients and neonatal death of one patient (1.2%). Similarly, a study given by Cheung et al. described fetal outcomes that involved low birth weight (<2.5 kg) consisting of 57 (60%) patients, neonatal (NICU) admission 51 (54%) patients, APGAR<7 at 5 mins involving 6 (6%) patients [27]. Additionally, Nischintha et al. reported fetal outcomes that involved Low Birth Weight (<2.5 kg) consisting of 31 (85.5%) patients, neonatal (NICU) admission involving 19 (52.8%) patients, and APGAR<7 at 5 mins including two (5.3%) patients [24]. In conclusion, the most common fetal outcome reported was low birth weight.

The objective of the study was to measure the accuracy of spot urinary albumin creatinine ratio with respect to 24-h UA for diagnosis of proteinuria in antenatal women with PE and concluded that spot UACR has more specificity than sensitivity along with a high negative predictive value for detection of proteinuria and hence can be used for diagnosis of proteinuria in women with PE but had certain limitations of the study consisting of small sample size, and shorter duration of the study period. Large multi-centric study groups with RCTs are required for reliable study outcomes along with long-term follow-up of patients.

## Conclusions

A challenging task for the obstetrician is using 24-h UA estimate to diagnose proteinuria and its link with fetomaternal outcomes and problems in PE. This gold standard procedure has been well associated with alternative testing techniques including spot UACR. The study concluded that spot UACR has more specificity than sensitivity along with a high negative predictive value for the detection of proteinuria and hence can be used for the diagnosis of proteinuria in women with PE. This study also observed that significant proteinuria was associated with an increased rate of induced labor and cesarean section in patients. Additionally, it was observed that significant proteinuria was associated with lower mean gestational age at the time of delivery, lower birth weight, and increased rate of IUFD.

Hence, spot UACR is a reliable, faster, and accurate method for the detection of proteinuria in PE and can be used for early diagnosis and timely management of women with PE which can help in reducing maternal and fetal mortality and morbidity. However, in low-resource nations like India, the price of UACR has been a deciding issue in its use, and the use of 24-h urine protein continues to be the deciding element in the diagnosis of PE.
